# Selected Predictors of COVID-19 Mortality in the Hospitalised Patient Population in a Single-Centre Study in Poland

**DOI:** 10.3390/healthcare11050719

**Published:** 2023-03-01

**Authors:** Joanna Jaskolowska, Elzbieta Balcerzyk-Barzdo, Agnieszka Jozwik, Tomasz Gaszynski, Pawel Ratajczyk

**Affiliations:** 1 Maria Sklodowska-Curie Provincial Specialist Hospital, 95-102 Zgierz, Poland; 2 Department of Anaesthesiology and Intensive Care, Medical University of Lodz, 90-153 Łódź, Poland

**Keywords:** COVID-19, CRP, D-dimer, ferritin, procalcitonin, LDH, RDW-CV, RDW-SD, clinical predictors of mortality

## Abstract

**Background**: The correct analysis of COVID-19 predictors could substantially improve the clinical decision-making process and enable emergency department patients at higher mortality risk to be identified. **Methods**: We retrospectively explored the relationship between some demographic and clinical factors, such as age and sex, as well as the levels of ten selected factors, namely, CRP, D-dimer, ferritin, LDH, RDW-CV, RDW-SD, procalcitonin, blood oxygen saturation, lymphocytes, and leukocytes, and COVID-19 mortality risk in 150 adult patients diagnosed with COVID-19 at Provincial Specialist Hospital in Zgierz, Poland (this hospital was transformed, in March 2020, into a hospital admitting COVID-19 cases only). All blood samples for testing were collected in the emergency room before admission. The length of stay in the intensive care unit and length of hospitalisation were also analysed. **Results**: The only factor that was not significantly related to mortality was the length of stay in the intensive care unit. The odds of dying were significantly lower in males, patients with a longer hospital stay, patients with higher lymphocyte levels, and patients with higher blood oxygen saturation, while the chances of dying were significantly higher in older patients; patients with higher RDW-CV and RDW-SD levels; and patients with higher levels of leukocytes, CRP, ferritin, procalcitonin, LDH, and D-dimers. **Conclusions**: Six potential predictors of mortality were included in the final model: age, RDW-CV, procalcitonin, and D-dimers level; blood oxygen saturation; and length of hospitalisation. The results obtained from this study suggest that a final predictive model with high accuracy in mortality prediction (over 90%) was successfully built. The suggested model could be used for therapy prioritization.

## 1. Introduction

In Wuhan, a town in China, cases of “unknown viral pneumonia” have been observed since December 2019. A pandemic outbreak of COVID-19 (Coronavirus Disease 2019) was declared on 11 March 2020 by the World Health Organization [1]. A total of 539,893,858 COVID-19 cases had been reported to the WHO by mid-2022, including 6,324,112 deaths. Although more than two-thirds of infected patients had mild-to-moderate symptoms, the remaining number required hospital therapy [2]. The analysis of the clinical prognostic factors of COVID-19 mortality could be beneficent for high-risk-patient treatment. The worsening of prognosis has been connected with demographic factors (age and sex) and comorbidities such as cardiovascular disease, diabetes, and obesity [3]. In patients suffering from COVID-19, abnormal values of hematologic parameters (lymphocytopenia), coagulation (D-dimer), and inflammation (especially CRP, PCT, and ferritin levels) are emerging, but according to the authors cited, data are deficient and seem to require validation [4,5]. However, other large clinical trials and complex data analyses using machine learning strategies show that the usefulness of the above-discussed laboratory tests as predictors of COVID-19 severity and mortality is increasingly better known and documented [6,7,8,9]. The early determination of correctly selected predictors of COVID-19 mortality is an important matter in preventing patient death.

### Aim of the Study

Our study investigated whether there was a statistical difference in the values of all selected predictors of COVID-19 mortality between survivors and non-surviving patients admitted to the COVID-19 hospital in Zgierz, Poland (the hospital was converted, during the pandemic, to a medical facility accepting COVID-19 patients only). The second aim was to attempt to construct a predictive model with high accuracy in mortality prediction in this disease. The selection of the most appropriate predictors for assessing clinical prognosis could prospectively stratify the risk of dying in hospital due to COVID-19. There are very limited studies [10,11] of predictors of COVID-19 mortality in the hospitalised patient population from Poland; previous papers only present predictors of COVID-19 mortality and admission to the ICU or examine the risk of mechanical ventilation, so they do not exactly correspond to our study. We believe that the knowledge of clinical predictors of COVID-19 mortality in patient populations from different parts of the world could contribute to better treatment and prevention of patient death.

## 2. Materials and Methods

### 2.1. Study Protocol

All units of a specialist hospital in Zgierz (Poland), by decision of the Ministry of Health, were converted, in March 2020, into a hospital admitting COVID-19 cases only. The management of the hospital, the head of the emergency department of Provincial Specialist Hospital in Zgierz, Poland, and the Medical University of Lodz ethics committee approved this study (protocol No. RNN/03/22/KE; 11 January 2022; head: prof. J. Drzewoski). When performing this study, the guidelines outlined in the Declaration of Helsinki were followed. As it was an observational, retrospective study, and after consulting the ethics board, it was decided that patient consent for treatment was considered as consent for the evaluation of laboratory test results in this study.

### 2.2. Study Population

No additional intervention was performed except for standard treatment. A total of 150 patients consecutively reporting to the ER were included in our retrospective study. All test samples were collected at admission to the hospital in the emergency room. According to the inclusion criteria, all subjects were adults (≥18 years old) with established COVID-19 infection. COVID-19 diagnosis was confirmed by means of SARS-CoV-2 detection in respiratory specimens using real-time RT-PCR methods. According to the exclusion criteria, five patients with incomplete laboratory analyses were not included in the study. Essential clinical and laboratory data for this study were sourced through hospital electronic medical records and transferred to pre-planned case report forms. Confidential patient information was not used. The age and sex of the patients were recorded for research purposes. Blood samples for testing were collected in the emergency room prior to admission.

### 2.3. Recorded Data

Basic demographic and clinical factors were selected for analysis according to the empirical knowledge of the authors. In widely understood inflammation, there is an increase in the level of factors such as CRP, leukocytes, and procalcitonin. D-Dimers are commonly designated as indicators of thrombosis. Inflammation and blood clotting problems are common in COVID-19, which is why these factors were selected. The levels of ten selected factors, namely, CRP, D-dimer, ferritin, LDH, RDW-CV, RDW-SD, procalcitonin, blood oxygen saturation, lymphocytes, and leukocytes, were analysed. The dynamics of laboratory changes could be observed during hospitalisation, because laboratory tests were repeated. However, only the results obtained at admission were considered in this study. The length of stay in the intensive care unit and the length of hospitalisation were also analysed. Chest CT scans were performed in the emergency room for all patients. Their findings were not considered in this study, since chest CT scans were usually not repeated afterwards. Moreover, the question of analysing these results is a very complex problem that could be discussed by radiologists in a separate article. In our study, we presented a laboratory data comparison between survivors and non-surviving patients. The standards adopted for discharge from the hospital were euthermia for at least 3 days, significant improvement in computed tomography of both lungs, and improvement in the parameters of the respiratory system in general. Additionally, a throat swab taken twice within a 24 h interval for SARS-CoV-2 RNA virus had to be negative.

### 2.4. Statistical Analysis

The analyses were performed using programme SPSS, version 26. The dataset was verified for the presence of outliers. Firstly, the groups of survivors and non-survivors were compared in terms of age and sex. The statistical significance of the difference between survivors and non-survivors in terms of age was assessed with Student’s *t*-test for independent samples, since age was measured on an interval scale, and the two groups were compared. Patient sex and laboratory results out of the reference ranges were nominal variables; therefore, data analyses regarding sex and laboratory data analyses were based on Pearson’s chi-squared test for independence if no cells with an expected count of less than 5 were detected or on likelihood ratios in case of crosstab with expected frequencies of less than 5. Cramer’s V measures of associations were also calculated. Associations between potential predictors and mortality were analysed with logistic regression analysis. The analysis was performed in two stages. In the first stage, each predictor was separately analysed. In the second stage, the predictors that were detected as statistically significant in the preliminary analysis were analysed with logistic stepwise regression analysis with the forward entry method based on Wald statistics. This procedure allowed us to build a single predictive model based on numerous predictors with high accuracy in mortality prediction. In addition, a series of analyses based on single predictors followed by the stepwise method let us minimize the effect of associations between predictors. Finally, the predictors used in the final logistic regression model were analysed as predictors with a multilayer perceptron neural network model. All predictors were standardized. The training partition was based on 70% of cases. The test partition was based on 30% of cases. Batch training was performed, and the scaled conjugate gradient method was applied.

## 3. Results

In a hospital admitting COVID-19 patients only, 150 adult patients were included in the study. During hospitalization, 86 patients died, and 64 were discharged. The median age of the 150 patients was 70.0 years (IQR 63.0–78.75), ranging from 27 years to 95 years, and the statistical significance of the difference between survivors and non-survivors in terms of age was assessed with Student’s *t*-test for independent samples. The age difference between the death group and the discharged group was significant (*p* < 0.01). Most patients were male.

**Figure 1:**
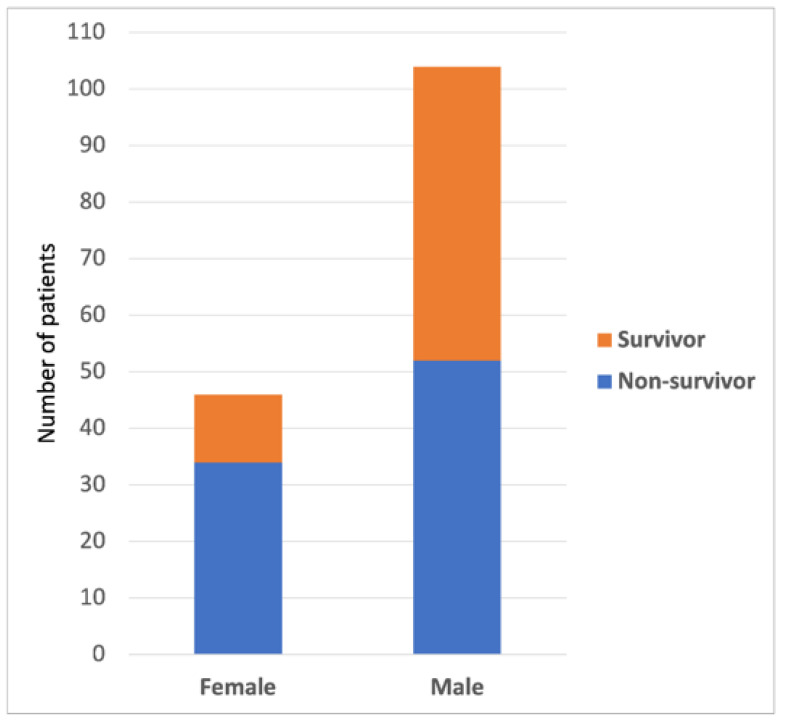
Gender mortality distribution.

The distribution of gender mortality is shown in Figure 1. Sex and laboratory data analyses were based on Pearson’s chi-squared test for independence if no cells with an expected count of less than 5 were detected or on likelihood ratios in case of crosstab with expected frequencies of less than 5. The number of men in the surviving group was significantly higher (81.3%) than in the non-surviving group (60.5%). The differences between survivors and non-survivors in terms of D-dimers, lymphocytes, leukocytes, procalcitonin, RDW-CV, and RDW-SD levels were statistically significant. In our study, the largest group of patients had lymphocyte levels below 10%. In this particular group, the mortality rate was above 70%. In the group with 10–20% lymphocyte levels, the mortality rate was 50%. With the increase in the number of lymphocytes, the survival rate increased. A comparison of the patient parameters of the two groups (leukocytes, D-dimers, and procalcitonin) showed that their levels were significantly lower in the surviving group than in the non-surviving group. Leukocyte levels below normal caused a mortality rate of 70%, while double the upper limit of normal levels only occurred in the group of non-survivors. A procalcitonin level above normal decreased the chance of survival. Our patients with levels above 0.5 ng/mL were the largest group, with a mortality rate of 78%. The average D-Dimer level did not exceed 3.5 μg/mL in survivors throughout the hospitalisation period. A procalcitonin level above normal decreased the chance of survival. The red cell volume distribution width CV (RDW-CV) and red cell volume distribution width SD (RDW-SD) were also significantly lower in the surviving group than in the non-surviving group. The norm for RDV-CV was defined using laboratory analysis as 11.5–14.5%. In the group of non-survivors, the IQR for RDV-CV was 13.9–17% with a median of 15.8%, while in the group of survivors, the IQR was 12.4–13.8% with a median of 13%. The norm for RDV-SD was determined using laboratory analysis as 36–47 fL. In the group of non-survivors, the IQR for RDV-SD was 45.5–56 fL with a median of 50.5 fL, while in the group of survivors, the IQR was 39.8–45 fL with a median of 42 fL. The proportions of out-of-norm results of LDH, ferritin, and CRP levels were significantly higher in the group of non-survivors (Table 1), except for the blood oxygenation saturation level, because only one patient in the entire sample of 150 patients had saturation in the reference range, i.e., at least 95%.

Data analysis was based on logistic regression analysis. Demographics and the exact values of laboratory results were analysed as potential predictors of mortality. Firstly, the predictive factors were separately analysed. The purpose of the analysis was to build a single predictive model with high accuracy in mortality prediction.

Table 2 summarizes the results of the analysis. All but one predictor were statistically significant. The only factor that was not significantly related to mortality was the length of stay in the intensive care unit. The odds of dying were significantly lower in males, patients with a longer hospital stay, patients with higher lymphocyte levels, and patients with higher blood oxygen saturation. The death occurrence rate was significantly higher in older patients; patients with higher RDW-CV and RDW-SD levels; and patients with higher levels of leukocytes, CRP, ferritin, procalcitonin, LDH, and D-dimers. To build a statistical model predictive of mortality, the stepwise regression method was used. Predictor entry was based on the values of Wald statistics. The results of the final model acquired after six steps are presented in Table 3.

Six predictors were included. As all of the predictive factors were separately analysed, the presence of two or more factors did not significantly affect the predicted mortality, and the occurrence of the third factor did not change the survival rate in relation to the examined factors. An increase of 1 year in patient age increased the chances of mortality by 1.171. An increase of 1 unit in the level of RDW-CV increased the chances of mortality by 3.11. An increase of 1 unit in the level of Procalcitonin increased the chances of mortality by 1.25. An increase of 1 unit in the level of D-dimers increased the chances of mortality by 1.12. An increase of 1 unit in saturation decreased the odds of mortality by 0.87. Hospital stay being prolonged by one day was associated with a decrease in the odds of mortality of 0.79. According to the Nagelkerke R^2^ index, the final model explained 81.5% of variance regarding mortality. It was capable of correctly classifying 91.9% of mortal cases and 90.6% of survivors. The overall percentage of correct classifications was equal to 91.3%.

**Figure 2:**
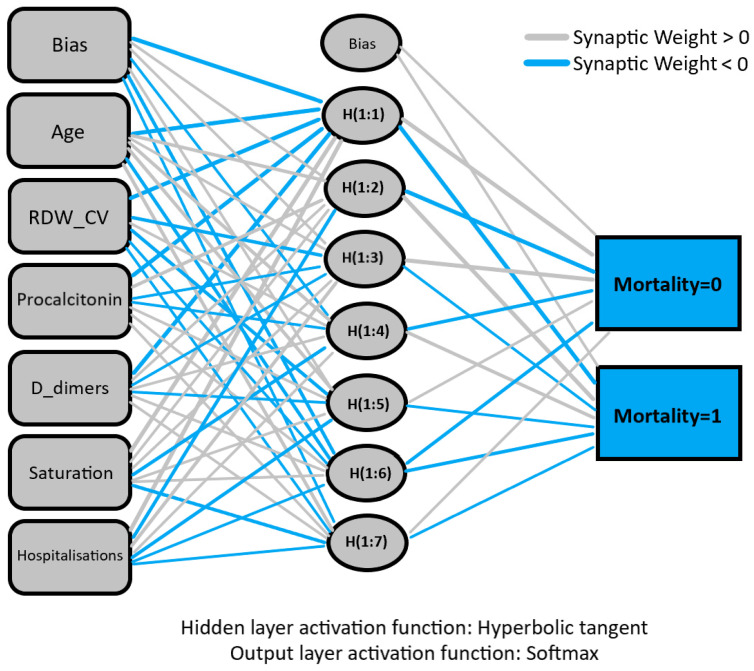
Network diagram acquired with multilayer perceptron multilayer model.

The predictors used in the final logistic regression model were analysed as predictors with a multilayer perceptron multilayer neural network model. Figure 2 depicts the network diagram.

**Figure 3:**
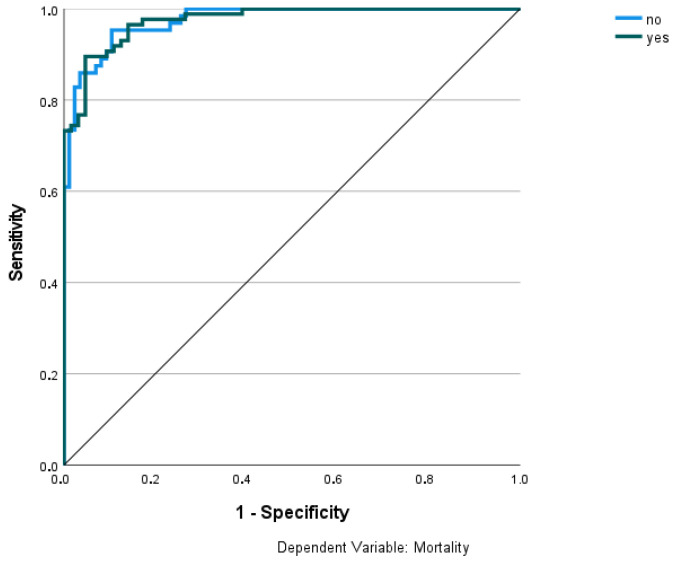
ROC curve based the predictions of multilayer perceptron multilayer model.

The training model predicted 88.3% of correct cases, 92.1% of mortal cases, and 83.3% of survivors. The testing model predicted 94.9% of correct cases, 95.7% of mortal cases, and 93.8% of survivors. Figure 3 depicts the ROC curve. The area under curve was equal to 0.975.

## 4. Discussion

Predictors of COVID-19 mortality represent an important and very topical matter in terms of COVID-19 treatment and prevention of patient death. The correct analysis of COVID-19 predictors could fundamentally improve the clinical decision process and enable faster diagnosis of patients at increased risk of death to be performed.

Our initial research on clinical predictors of mortality due to COVID-19 based on the analysis of data of 150 patients from Wuhan, China, included demographics, clinical characteristics, laboratory results, treatment options, and outcomes. Statistical analyses comprised continuous measurements as means (SDs) or as medians (IQRs), which were compared with Student’s *t*-test or Mann–Whitney–Wilcoxon test. Categorical variables were expressed as numbers (%) and compared with the χ^2^ test or Fisher’s exact test. Similar to our study, the differences in white blood cell counts, absolute values of lymphocytes, and levels of C-reactive protein (CRP) between the non-surviving and surviving groups were significant. A significant difference in age (older patients) between the death group and the discharged group (*p* < 0.001) was also observed [12]. In our study, the same number of patients was examined. We realized that it is impossible for a single-centre study to examine as many cases as in the work conducted by Khan et al. [13]. Their models were crafted using confirmed COVID-19 patients from 146 countries with 97,941 records of surviving patients and 5947 records of non-surviving patients. Their meta-analysis used ML algorithms, such as decision tree (DT), logistic regression (LR), random forest (RF), extreme gradient boosting (XGBoost), and K-nearest neighbour (KNN) and the DL model (containing six layers with ReLU and an output layer with sigmoid activation), to predict the mortality rate in COVID-19 cases.

The authors of [14] presented a mortality risk prediction model for COVID-19 (MRPMC) that uses patient clinical data at admission to stratify patients by mortality risk, which enables physiological deterioration and death to be predicted up to 20 days in advance. This ensemble model was built using four machine learning methods, including logistic regression, support vector machine, gradient boosted decision tree, and neural network. Factors were analysed, and among these, high-risk features had a positive association with mortality (e.g., male sex, D-dimer, and age), while low-risk features were negatively correlated with mortality (e.g., lymphocytes). Our results agree with the presented findings, except for the positive association between mortality and the male sex.

This is one of the first studies reflecting on “time-to-event” at admission and accurately predicting patient outcomes performed on the Indian population. Machine learning (ML) modelling was performed using the extreme gradient boosting (XGB) algorithm. Further, “time-to-event” using the Cox proportional hazard model was used and combined with XGB. Beyond India, further research is needed to calibrate the model when used in the other populations, such the US, Europe, Middle East, and Southeast Asia. The male gender has a hazard ratio of 1.72 (95% CI 1.06–2.85), signifying a higher risk of mortality in the male population [15], which is congruent with the other international studies [13,16,17,18,19,20] and is inconsistent with the results presented in our study.

In a systematic review and meta-analysis of prognostic factors, study [6], using the data presented as medians and interquartile ranges and calculating the means and standard deviations based on the formulas used by Wan et al., confirmed, similarly to our study, that older age was correlated with increased mortality rate in patients with COVID-19. Also similar to our patient population, the values of D-dimer, C-reactive protein (CRP), procalcitonin (PCT), ferritin, and lactate dehydrogenase (LDH) were significantly higher in non-surviving patients than in survivors; the levels of lymphocytes (LYM) were significantly lower in non-survivors than in survivors. The male sex was correlated with a higher death rate than the female sex, which has not been confirmed by us.

Predictive model usage could be beneficial to clinicians for tagging patients at increased risk of death. This could allow exceptional care to be provided to protect against death [21]. This study provided data on the demographic factors, and clinical and laboratory characteristics of hospitalised patients with COVID-19 at Provincial Specialist Hospital in Zgierz, M. Sklodowska-Curie, Poland. This study classified more than 50% of patients as severe cases (death of patients), and this figure differs from those reported in other studies [22,23]. As in previous studies [24,25,26], it was confirmed that some parameters observed at admission, such as older age or high LDH levels and leucocytosis, were associated with increased risk of death during hospitalisation. Yanez et al., using a Poisson mixed effects regression model, found that COVID-19 mortality rates were strongly associated with older age. Fatality rates vary significantly among different countries. Several factors may contribute to these differences, including the type of healthcare system, patient characteristics, and prevalence of diagnostic testing. Since the number of comorbid conditions (such as hypertension, diabetes, and obesity) steadily increases with age, this could be another logical explanation of the observed increased mortality in older patients. Additionally, changes associated with immunosenescence might explain the increased vulnerability to infection and the disproportionately high mortality due to COVID-19 in older patients [16]. In many diseases, including oncological diseases and infections, elevated levels of LDH are a factor of serious prognosis [27]. In this study, we did not statistically analyse the performed chest CT, because it was usually not repeated afterwards and chest CT scans show poor specificity for COVID-19 interstitial pneumonia [28]. Lung and tissue damage seen in patients with severe COVID-19 is believed to be associated with high levels of LDH [21]. The median age of our study population was 70 years, and the increase of 1 year in patient age increased the odds of mortality by 1.171. In our study, the number of men in the group of survivors was significantly higher (81.3%) than that in the group of non-survivors (60.5%). This was not confirmed by other authors, who reported that male patients with COVID-19 are more symptomatic and they show increased disease severity, higher complication rates, and consequently, higher mortality [29,30]. Works by different authors indicated a relationship between high risk of death and an increased level of D-dimer [31,32]. The above observation can be used as a marker in predicting mortality, as it was observed that patients who did not survive had significantly higher levels of D-dimer. Some investigators proposed the use of D-dimer blood levels in patient triage [33]. The latest large clinical studies confirmed the cited results concerning D-dimer level and elderly age [34] or blood oxygen saturation and CRP level [35]. In meta-analyses of the literature, as well as in individual works, many authors have reported, as we do, that patients suffering from COVID-19 with a poor prognosis have higher serum ferritin levels than patients with a good prognosis.

Ferritin has been identified as a signalling molecule and a direct mediator of the immune system. It is an “acute phase reactant” reflecting the degree of both chronic and acute inflammatory responses within the body. However, it is uncertain whether hyperferritinemia is a result or a mediator of inflammation. A higher level of ferritin indicates the activation of the monocyte–macrophage system, in which ferritin synthesis responds to changes in the status of cytokines at both the transcription and translation levels.

High ferritin values may serve as an important prognostic biomarker in the triage of patients with more severe COVID-19 disease and poor prognosis. However, in the presence of other comorbidities, serum ferritin should be interpreted with caution [36,37,38]. We showed that in the group of patients studied, the increase of 1 unit in the level of D-dimers increased the odds of mortality by 1.12. Similarly, one of the recent studies showed that blood oxygen saturation values below the normal range indicate a poor prognosis [32]. Our results confirmed that the chances of mortality were significantly lower in patients with higher blood oxygen saturation. An increase of 1 unit in saturation decreased the odds of mortality by 0.87. Inflammatory factors such as CRP and PTC observed in patients with high mortality during COVID-19 are becoming increasingly well documented. Many authors believe that bacterial infections that occur in patients with severe COVID-19 symptoms are directly related to high CRP and PTC levels [39]. High mortality in COVID-19 is also associated with elevated levels of CRP and PTC in blood serum. In our study, we confirmed that the odds of mortality were significantly higher in patients with elevated levels of CRP and procalcitonin. In addition, the increase of 1 unit in the level of procalcitonin increased the odds of mortality by 1.25. The predictive role of haematological parameters in foreseeing infection severity in patients with COVID-19 appears in a few reports. Haematological tests, with their low detection price and high automation rates, are easily accessible indicators of disease course and overall assessment in patients. Some haematological parameters transform significantly in COVID-19 patients. A significant drop in the lymphocyte count was noticeable in patients who had died compared with survivors [40]. Our laboratory results confirmed the above reports, as there were statistically significant differences between the survivors and the non-survivors in the number of lymphocytes in blood. Elevated RDW appears in many diseases associated with increased risk of disease outbreak and mortality; among different diseases connected with higher levels of RDW are pulmonary disease, sepsis, influenza, cancer, anaemia, chronic obstructive pulmonary disease, and diabetes [41]. Some authors point at the underestimation of significant increases in RDW as a risk indicator in patients with severe COVID-19 [42,43]. The values of the RDW-CV and RDW-SD parameters in patients with more severe disease were significantly higher than in patients with less severe disease [40]. Our work shows that the odds of dying were significantly higher in patients with higher RDW-SD and RDW-CV levels. Additionally, it was found that the increase of 1 unit in the level of RDW-CV increased the odds of mortality by 3.11. Data of the median length of ICU stay in China, Singapore, and the US indicate around seven days for COVID-19 survivors and eight days for non-survivors, with shorter stays being reported in the UK [44]. In our study, the only factor that was not significantly related to mortality was the length of stay in the intensive care unit, but the odds of mortality were significantly lower in patients with a longer hospital stay. Hospital stay being extended by one day was associated with a decrease in the odds of mortality of 0.79. Some studies reported that COVID-19 patients with poor prognosis had significantly elevated levels of D-dimers, CRP, LDH, and lymphocytopenia at admission to the hospital [45]. Reduced lymphocyte counts, and elevated CRP, PCT, and LDH levels were predictors of death, increasing the risk of death by 3–4 times. Other studies attempted to develop a survival risk prediction model to foresee progression to severe or critical disease, which is consistent with our study and confirms that patients with a higher D-Dimer value are at higher risk of death [46,47]. We emphasize that all data were collected at admission to the hospital in the emergency room from patients at Provincial Specialist Hospital in Zgierz, Poland. Selected tests were performed, obviously relying on data partially known from the literature. The tests that were relatively effective in imaging the patient conditions were selected. In addition, these were tests that could be performed under pandemic conditions, with limited human and financial resources. All the patients in our hospital were symptomatic, with most being severely and even critically so, with a mortality rate as high as 57.3%. Additionally, asymptomatic and mild patients were not admitted to the hospital, so the results may not be representative of all types of patients, especially asymptomatic and mild patients. In addition, the values of blood oxygen saturation, D-dimers, LDH, and CRP were out of the reference ranges in most patients of both groups, which made the analysis insensitive to detecting statistically significant relationships between these variables and mortality, as presented in Table 1. The final model presented in Table 3 may be biased by correlations among the included predictors. The obtained results may differ between different patient populations. They could help predict severity and mortality in COVID-19, which is very useful in clinical application. The authors hope that the data analysis on the Polish population expands the knowledge about predictors of COVID-19 mortality in patients at admission to the hospital emergency room. It is also the significance of this study.

### Limitations

We recognize that this study has some limitations. It is a retrospective, single-centre study, and this explains the limited number of analysed cases. However, even a small population of examined patients increases our knowledge about the new problem, which is the prevention of death in COVID-19. Additionally, in our study, only patients in serious conditions were admitted to the hospital, so the results may not be representative of all types of patients, particularly asymptomatic and mild patients.

## 5. Conclusions

The emergence of COVID-19 has caused widespread concern and has ruined public health security worldwide. The predictors of lethal COVID-19 outcome were analysed. Six potential mortality predictors were included in the final model: age, RDW-CV, procalcitonin and D-dimer levels, blood oxygen saturation, and length of hospitalisation. Statistically significantly higher values of the analysed parameters were demonstrated in non-survivors. The results obtained in this study allowed us to build a definitive predictive model with high accuracy in mortality prediction (over 90%). More research is needed to analyse the cause of the statistically significantly higher values of the analysed parameters. The reason why, in the literature, the male sex has been correlated with a higher death rate than the female sex, which has not been confirmed by us, remains unclear. This may result from the pathophysiology of the disease and the receptor representation for the virus possessed by men, and it may also result from differences in patient populations from different parts of the world; therefore, it requires clarification in other studies in our part of Europe. This is necessary to ensure effective treatment of COVID-19 infection and reduce the death rate. The authors understand that due to the small dataset size, the results are not generalizable, but we hope that the suggested model can be used for therapy prioritization. It is important to identify risk factors already at the stage of admitting a patient with an early therapeutic approach, as it can significantly improve survival rates.

## Figures and Tables

**Table 1:** Demographics and percentage of patients with laboratory results out of the reference range of survivors and non-surviving patients.

	All Patients	Survivors	Non-Survivors	*p*-Value	d/V
Demographics and laboratory characteristics					
Age, years	69.64 (63–78.75)	62.66 (53.75–71.0)	74.84 (68.25–83.0)	*p* < 0.001	11.51
Sex	..	..	..	0.014	
Female	46 (30.7%)	12 (18.8%)	34 (39.5%)	0.006	0.22
Male	104 (69.3%)	52 (81.3%)	52 (60.5%)		
Saturation	149 (99.3%)	63 (98.4%)	86 (100%)	0.191	0.09
D-dimers	134 (89.3%)	51 (79.7%)	83 (96.5%)	0.001	0.27
LDH	143 (95.3%)	61 (95.3%)	82 (95.3%)	0.992	0.01
Lymphocytes	118 (78.7%)	43 (67.2%)	75 (87.2%)	0.003	0.24
CRP	143 (95.3%)	60 (93.8%)	83 (96.5%)	0.431	0.06
Leukocytes	61 (40.7%)	15 (23.4%)	46 (53.5%)	*p* < 0.001	0.30
Procalcitonin	56 (37.3%)	12 (18.8%)	44 (51.2%)	*p* < 0.001	0.33
Ferritin	150 (100%)	64 (100%)	86 (100%)	-	
RDW-CV	46 (30.7%)	4 (6.3%)	42 (48.8%)	*p* < 0.001	0.46
RDW-SD	40 (26.7%)	3 (4.7%)	37 (43.0%)	*p* < 0.001	0.43

Note. d—Cohen’s d effect size measure; V—Cramer’s V effect size measure.

**Table 2:** Analysis of relationships between potential predictors and mortality.

Predictor	OR {95% CI}	Wald	*p*-Value	R^2^
Sex (male patient)	0.35 [0.16; 0.76]	7.17	0.007	0.067
Age	1.10 [1.06; 1.14]	25.14	0.001	0.290
Intensive care unit stay	3.30 [0.89; 12.22]	3.19	0.074	0.034
Length of hospitalisation	0.86 [0.82; 0.92]	24.75	0.001	0.285
RDW-CV	2.45 [1.80; 3.33]	32.89	0.001	0.452
RDW-SD	1.25 [1.16; 1.35]	31.42	0.001	0.417
Lymphocytes	0.92 [0.88; 0.96]	13.59	0.001	0.144
Leukocytes	1.14 [1.07; 1.22]	14.68	0.001	0.187
CRP	1.04 [1.00; 1.08]	4.32	0.038	0.040
Ferritin	1.01 [1.01; 1.01]	9.48	0.002	0.125
Procalcitonin	1.18 [1.01; 1.39]	4.20	0.040	0.076
LDH	1.00 [1.00; 1.00]	8.46	0.004	0.117
D-dimers	1.14 [1.02; 1.27]	5.38	0.020	0.122
Saturation	0.81 [0.72; 0.91]	11.84	0.001	0.189

ORs—odds ratio; Wald—Wald statistics; R^2^—Cox and Snell explained variance index.

**Table 3:** Potential predictors of mortality included in the final model.

Predictor	OR	Wald	*p*
Age	1.17 [1.08; 1.27]	16.13	0.001
RDW-CV	3.11 [1.86; 5.20]	18.63	0.001
Procalcitonin	1.25 [1.05; 1.48]	6.18	0.013
D-dimers	1.12 [1.02; 1.23]	5.65	0.017
Saturation	0.87 [0.76; 1.00]	3.72	0.054
Length of hospitalisation	0.79 [0.70; 0.88]	17.27	0.001

Note. According to the Nagelkerke R^2^ index, the final model explained 81.5% of variance regarding mortality. It was capable of correctly classifying 91.9% of mortal cases and 90.6% of survivors. The overall percentage of correct classifications was equal to 91.3%. OR—odds ratio; Wald—Wald statistics; *p*—statistical significance

## Data Availability

Data are available upon demand from the authors.
